# A cluster-randomized controlled trial to assess the effectiveness of using 15% DEET topical repellent with long-lasting insecticidal nets (LLINs) compared to a placebo lotion on malaria transmission

**DOI:** 10.1186/1475-2875-13-324

**Published:** 2014-08-16

**Authors:** Onyango Sangoro, Elizabeth Turner, Emmanuel Simfukwe, Jane E Miller, Sarah J Moore

**Affiliations:** Ifakara Health Institute, Box 74, Bagamoyo, Tanzania; Disease Control Department, London School of Hygiene and Tropical Medicine, Keppel Street, London, WC1E 7HT UK; Department of Biostatistics and Bioinformatics and Duke Global Health Institute, Duke University, Duke Box 2721, Durham, NC 27705 USA; Population Services International, Dar es Salaam, Tanzania; Department of Health Interventions, Swiss Tropical and Public Health Institute, Socinstrasse, 57, CH-4002 Basel, Switzerland; University of Basel, Petersplatz 1, 4003 Basel, Switzerland

## Abstract

**Background:**

Long-lasting insecticidal nets (LLINs) have limited effect on malaria transmitted outside of sleeping hours. Topical repellents have demonstrated reduction in the incidence of malaria transmitted in the early evening. This study assessed whether 15% DEET topical repellent used in combination with LLINs can prevent greater malaria transmission than placebo and LLINs, in rural Tanzania.

**Methods:**

A cluster-randomized, placebo-controlled trial was conducted between July 2009 and August 2010 in a rural Tanzanian village. Sample size calculation determined that 10 clusters of 47 households with five people/household were needed to observe a 24% treatment effect at the two-tailed 5% significance level, with 90% power, assuming a baseline malaria incidence of one case/person/year. Ten clusters each were randomly assigned to repellent and control groups by lottery. A total of 4,426 individuals older than six months were enrolled. All households in the village were provided with an LLIN per sleeping space. Repellent and placebo lotion was replaced monthly. The main outcome was rapid diagnostic test (RDT)-confirmed malaria measured by passive case detection (PCD). Incidence rate ratios were estimated from a Poisson model, with adjustment for potential confounders, determined *a priori*. According-to-protocol approach was used for all primary analyses.

**Results:**

The placebo group comprised 1972.3 person-years with 68.29 (95% C.I 37.05-99.53) malaria cases/1,000 person-years. The repellent group comprised 1,952.8 person-years with 60.45 (95% C.I 48.30-72.60) cases/1,000 person-years, demonstrating a non-significant 11.44% reduction in malaria incidence rate in this group, (Wilcoxon rank sum z = 0.529, p = 0.596). Principal components analysis (PCA) of the socio-economic status (SES) of the two groups demonstrated that the control group had a higher SES (Pearson’s chi square = 13.38, p = 0.004).

**Conclusions:**

Lack of an intervention effect was likely a result of lack of statistical power, poor capture of malaria events or bias caused by imbalance in the SES of the two groups. Low malaria transmission during the study period could have masked the intervention effect and a larger study size was needed to increase discriminatory power. Alternatively, topical repellents may have no impact on malaria transmission in this scenario. Design and implementation of repellent intervention studies is discussed.

**Trial registration:**

The trial was registered ISRCTN92202008 - http://www.controlled-trials.com/ISRCTN92202008

**Electronic supplementary material:**

The online version of this article (doi:10.1186/1475-2875-13-324) contains supplementary material, which is available to authorized users.

## Background

In the past decade, considerable financial and political resources have been mobilized for malaria control [1]. This has in turn led to extensive coverage and use of existing control tools, like long-lasting insecticidal nets LLINs and indoor-residual spraying (IRS) [1]. Implementation of these highly effective vector control tools has resulted in substantial decrease in malaria transmission, morbidity and mortality [2–4]. Despite both extensive coverage and use, the sole use of these tools have not and will not be able to eliminate malaria in all malaria endemic regions [5]. Because LLINs and IRS target mainly indoor biting and indoor resting vectors their implementation may select for outdoor resting and biting vector populations that often become dominant, so that even though there is a diminished malaria transmission as a result of extensive LLINs and IRS use, there is likely to be a larger proportion of this residual transmission occurring outdoors compared to indoors [6].

Increased urbanization and rural electrification programmes have also had an impact on malaria transmission dynamics. As a result of this, individuals stay up later in the evenings than they usually would in a situation where electricity was not available [7], and are, therefore, exposed to potentially infective mosquito bites for longer.

With the renewed push for malaria elimination [8], it is evident that new tools need to be developed to augment existing vector control tools to achieve this goal. Topical repellents provide excellent personal protection [9] and could potentially be used to complement LLINs for additional protection from residual transmission [5]. Several studies demonstrated that topical repellents offer additional protection from malaria transmission either when used alone, or in combination with LLINs, in areas with high early evening and outdoor malaria transmission [10–12].

This study assessed the potential additional benefit of using topical repellents in combination with LLINs compared to using only LLINs on early evening malaria transmission in a rural community in Kilombero valley, south-west Tanzania.

This community mainly relies on subsistence farming of rice, which provides for a large breeding site for both malaria vectors and nuisance biting mosquitoes [13]. It is customary that the community in the study area cook outdoors in the early evenings, a situation that is likely to expose them to mosquito bites and potential malaria transmission. Rural development is also rapidly taking place in this study area. As a result, many members of the community usually gather in the early evening and stay late into the night at local entertainment spots that are springing up in the study area owing to rural electrification programmes, thereby increasing the potential of malaria transmission at these times. A recent report estimates a malaria incidence rate of 0.67 cases/person/year confirmed by rapid diagnostic test (RDT) from passive case detection at a local clinic between December 2012 and July 2013 (Jabari Mohammed Namamba, pers. comm.).

In the past two decades, extensive malaria intervention programmes have taken place in this area, and it is therefore expected that the community be highly sensitized on malaria transmission and control methods [14–17]. There is high LLIN use in the study area [18]. Repellent awareness and knowledge as assessed using a Knowledge, Attitude and Practice (KAP) baseline questionnaire at the inception of the clinical trial determined that this community did not use topical repellents as a mosquito control tool. Awareness and availability were reported as the major reasons for not using topical repellents [Sangoro O, Sarah M, Ann HK, Sarah M: **Feasibility of repellent use in a context of increasing outdoor transmission: A Qualitative study in rural Tanzania, submitted to Malaria Journal for publication**].

The major malaria vector in the study area is *Anopheles arabiensis*[19], which has been shown to exhibit elastic feeding behaviour depending on the availability and location of the host [6] and is known to exhibit early evening biting [20]. The dominance of this vector in this area is also likely to be the result of extensive LLIN use in the study area [21, 22].

A field study conducted in the study area to determine the efficacy of this repellent (15% DEET) against *An. arabiensis* demonstrated >80% protection from bites over four hours of mosquito collection [19]. Therefore, 15% DEET was considered appropriate to provide protection against early evening biting.

This study area was chosen because there are no studies that have been conducted to assess the additional benefits of topical repellents to LLINs in malaria control in East Africa, although this technology has been shown to work elsewhere in sub-Saharan Africa [23, 24]. Also the vectors present in the area, *An. arabiensis*, exhibit early evening biting [20], a trait that made the use of repellents in the early evening ideal in this area. Therefore, even though extensive employment of current control tools will lower malaria transmission in this area, its is likely that residual transmission will continue occur at times when the effectiveness of these tools is diminished, like outdoors in the early evenings and mornings, [6] and will require supplementary tools that target this scenario.

Therefore, it was hypothesized that combined use of LLINs and topical repellents in this community would have a greater impact on malaria transmission in the early evening compared to sole use of LLINs.

## Methods

### Study area

The study was carried out in Mbingu village, Ulanga district, situated 55kms west of Ifakara town at 8.195°S and 36.259°E. At the time of the study inception, (July 2009), the village was estimated to have 7,609 inhabitants [25]. There is moderate malaria transmission in the study area, with peak transmission occurring in the months of May and June after the long rains. The village experiences an annual rainfall of approximately 1,200-1,800 mm and an annual temperature range of between 20°C and 32.6°C. The village borders an extensive field cleared for rice irrigation, which provides an ideal breeding site for malaria vectors [13].

### Sample size rationale

The only available data from the study area were community reported fever incidence rate estimates of 3.2 cases/person/year for children under the age of five years [26]. Assuming fever rates in children under five years are higher than the rest of the population, and that not all fevers reported are caused by malaria, a rate of one malaria case/person/year was used to calculate the sample size needed for this study. Available reports also indicated that 30% of mosquito bites occurs in the early evening [20]. Therefore, assuming that mosquitoes have an equal probability of carrying sporozoites regardless of time of night, it was assumed there was a potential 30% malaria transmission occurring in the early evenings. Expecting that repellents would reduce 80% of this potential 30% early evening transmission, as observed from the field study [19], it was reasoned that repellents would reduce the overall transmission of malaria from one case/person/year to 0.76 cases/person/year. Using the methods of Hayes *et al.*[27] for sample size calculation for cluster randomized trials, it was estimated that to observe this treatment effect (24%), with 90% power at the two-tailed 5% significance level, 10 clusters of 47 households with five members each was required per treatment group. A coefficient of variation (k) of 0.20 was used based on published recommendations as the inter-cluster variation could not be estimated [28].

### Household recruitment

Households were recruited into the study in two phases. In phase one, the study investigators and field team visited the study village for reconnaissance and introduction to the community leaders and members in December 2008. A week later, the study team returned to the study village and aided by community leaders, identified the centre of the village. Here, the field team spun a ballpoint pen and visited all the households that the writing end of the pen pointed to with the intention of recruiting all consenting households into the study. After all households in this direction had been exhausted, the field team went back to the village centre and spun the pen to choose the next direction in which to visit the households. If the pen pointed in the direction where the households were already visited, then, the pen was spun again until a new direction was identified. This progression was repeated until approximately, 1,000 households had been visited and recruited. The village had 2,000 households [25] and, therefore, by visiting and potentially enrolling at least 50% of the households, the study team were confident that they had captured a representative sample of households in the study area.

### Enrolment of households into the study

During the household recruitment visits, each household head was informed of the purpose of the visit. They were educated on the objectives, risks and benefits of the study to their household and the community. They were encouraged to ask questions and after all their concerns had been addressed, they were asked if they were willing to participate in the study. If willing, each household head was asked to sign a written informed consent form, confirming their participation and that of all household members. As data was being collected at the household level, only the household head was asked for informed consent. It was assumed that once that household head gave consent then all household members would likely comply with repellent use following instructions of the household head as the authority in each household. A structured questionnaire on the socio-economic status (SES) of the household and knowledge, attitude and practice (KAP) in relation to malaria and repellents was then administered [Sangoro O, Sarah M, Ann HK, Sarah M: **Feasibility of repellent use in a context of increasing outdoor transmission: A Qualitative study in rural Tanzania, submitted to Malaria Journal for publication**]. The GPS coordinate of the household enrolled was then recorded using a handheld GPS receiver (Garmin eTrex Legend® H). These coordinates were then plotted using Arc GIS software (Arc GIS 9.0, ESRI, UK), to generate a map of all the households enrolled in the study area.

### Second phase of household recruitment, household enrolment and cluster generation

**Figure 1:**
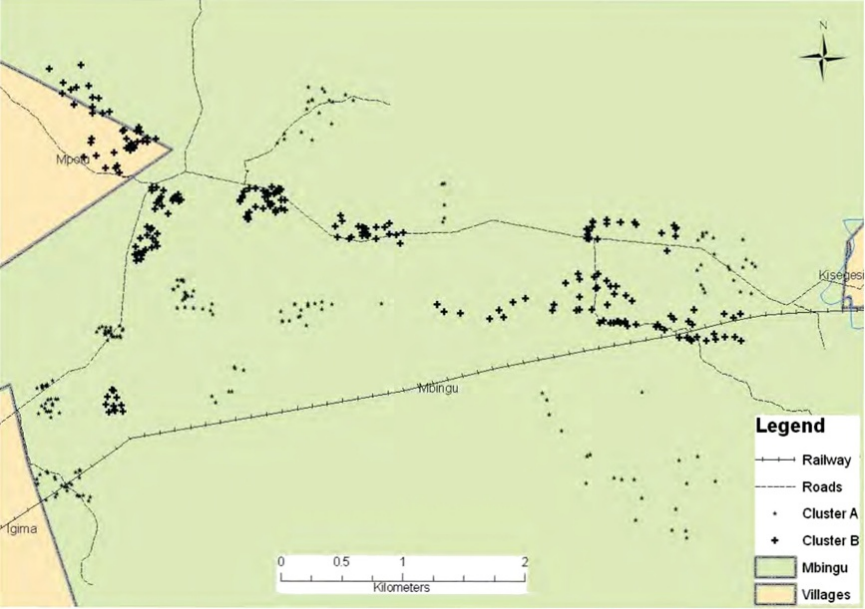
**Map of households recruited into the trial in the study village.**

In phase two, the map generated during the first phase of recruitment was used to delineate 20 clusters of households each while ensuring a buffer zone of 200 metres between clusters to prevent diversion of mosquitoes from the intervention group to the control group. As a result of creation of this buffer area, some households that had been recruited in the first phase fell within this 200 metre buffer area. These households were excluded from the study during this second phase of recruitment. Therefore, even though about 1,000 households were recruited in the first phase, more households needed to be recruited in the second phase as a result of loss of households within the buffer area. These households were excluded because they would have potentially confounded the outcome of the study in case of diversion of mosquitoes. All households within the buffer area were issued with an LLIN per sleeping space to protect them from potentially greater than normal bites from diverted mosquitoes. In practice, the second phase of recruitment proceeded as follows: The field team visited the 20 clusters, using the household considered to be at the centre of these clusters (identified from the Arc GIS map), as the starting point. The household head of the central household in the cluster was informed of the purpose of the visit. If the household had been enrolled during the first phase of household recruitment, then the field team issued an LLIN for every sleeping space, stapled a unique identifier number on the door frame and moved to the next nearest household. If the households had not been enrolled, the household head was informed of the objectives, risks and benefits of the study, enrolled on written informed consent, provided with a unique household identifier and LLINs for each sleeping space, and a SES and KAP questionnaire administered. This progression was repeated until 47 households close together were enrolled to form a single cluster. All 47 households in each of the 20 clusters were enrolled in this manner. The newly enrolled households that did not appear on the map generated in the first phase of recruitment were plotted and the map updated to produce the final map of households recruited into the study (Figure 1).

Clusters were used as the unit of randomization for three reasons: 1) since the intervention would be applied to a community, if proven to be effective, 2) to limit contamination of treatments between households, and 3) to avoid diversion of mosquitoes from individuals who used repellents to those who did not use repellent within the same household of from households using repellents to households that used the placebo, thereby putting non-repellent using individuals and households at a potentially higher risk of contracting malaria [29, 30].

### Eligibility criteria

All households were eligible to be recruited into the trial and no household was excluded on the basis of household structure, asset or livestock ownership. All individuals older than six months of age were eligible to be recruited into the trial. This age cut-off was used because re evaluation of DEET insect repellent [31] estimated the margin of exposure (MOE) in children less than six months to be less than 100. Margin of exposure is defined as the ratio of dose of DEET used daily to the no observed effect level dose recommended by regulation agencies, which usually consider doses, which result in MOEs of less than 100, unacceptable. Based on this risk assessment, use of DEET was not recommended for children under six months [32].

### Randomization of clusters to treatments

All the 20 clusters in the map (Figure 1) were assigned numbers 1 to 20, starting from the left hand side to the right. The cluster numbers were then written down on small pieces of paper, which were placed in a bowl. The principal investigator (PI) and project leader (PL) then drew the pieces of paper from the bowl one at a time. Two three digit numbers (258 and 305) were used to classify clusters in to two groups. The first cluster number to be drawn was assigned treatment 258 and the second cluster number assigned treatment 305. This progression was repeated until all the clusters had been assigned to one of the two groups.

### Blinding

The repellent and placebo lotion smelt and felt the same and were placed in identical tubes, distinguishable only by the two three-digit numbers known only to the independent code keeper (SC Johnson and Sons). However, the PI and PL had previously conducted efficacy test of these two treatments [19], and could identify the repellent and placebo from the results of this study. Therefore, it was only the field team, study statistician and study participants who were blinded in this study. Blinding was broken after analysis.

### Repellent issuance, application and compliance

In June 2009, the field team visited all households enrolled in the study to distribute treatments to study participants. The treatments, (15% DEET and placebo), both formulated as a pourable lotion that is applied by hand, were supplied by SC Johnson, Racine, USA, and packaged in 100 ml plastic tubes. During this visit, the field team informed the household members on how to apply the treatments provided on exposed areas of the body. They also advised the participants not to apply the treatments on open wounds, eyes, mouth and areas with mucous membranes. The repellent lotion was applied at an approximate rate of 0.002 mg DEET/cm^2^, the quantity of repellent that prevented >80% mosquito bites for 4 hours in a controlled environment and in the study area [19]. Even though a repellent with a higher concentration would have provided greater protection, the Tanzania National Institute of Medical Research ethical approval board did not allow the use of a repellent that had more than 15% DEET due to safety concerns, despite the initial request of the PI to use 30% DEET and submission of detailed experimental justification and dossier of safety data justifying the use of a higher concentration.

The participants were issued measuring caps, with amounts of repellent required for adults (7mls) and children below 12 years (3mls) marked on the cap. Each tube held 100mls of repellent. Therefore, two tubes were considered enough to last an adult one month, i.e. if they applied the recommended dosage of 7 mls per day, while one tube was enough to last a child < 12 years for one month, if they used 3mls per day. Children > 12 years were advised to use up to 7mls a day, and were therefore issued with 2 tubes for the month. All the tubes issued per cluster and households were identical, and it is possible that the household members shared a single tube of repellent until it ran out. As all households member were issued with enough treatment to last them month, either 15% DEET repellent lotion or placebo, and dosages for adults and children had been marked out, it was assumed that sharing of repellents within the household would have no effect on the outcome as long as there was daily compliance to the recommended dose by the participants. The amounts recommended were adjusted to accommodate for individuals with greater than average body mass as it was determined from semi-field and field experiments that an average sized volunteer required 6 mls [19]. This amount was, therefore, adjusted upwards by an extra millilitre. The community members were instructed to apply the repellent at dusk (1800 hrs) and to reapply it if they felt any mosquito bites or remained active for more than four hours after sunset.

Compliance to lotion use (both repellent and placebo) was assessed by the field team visiting the enrolled households at the beginning of each subsequent month (monthly monitoring surveys) to issue new tubes of repellent and placebo lotion. Therefore compliance was assessed on a monthly basis using a short structured questionnaire, where the household head or an adult household member, was asked if all household members had used the repellents and reasons for non-compliance where relevant. However, as self-reported data are unreliable, the number of repellent/placebo tubes issued every month was also recorded as a secondary measure of compliance, to determine if there was a difference in the number of tubes issued in each month per treatment group. Data on use of LLINs the previous night, malaria infection, recalled febrile illness and visit to the health centre during that month was also collected. If, during these monthly monitoring surveys, the household head or any other adult household member was not available to answer the questionnaire on compliance, the field team visited that particular household daily for seven consecutive days. If still no household member able to take the monitoring survey was available during these repeated visits, then that household, and all it members, was excluded from the calculation of person-time for that month.

In addition to the compliance, malaria and recalled febrile illness data collected during each month of the study period, an after study questionnaire was administered at the close of the study to assess the participants’ knowledge, attitudes and practice in relation to repellents. These results are reported elsewhere [Sangoro O, Sarah M, Ann HK, Sarah M: **Feasibility of repellent use in a context of increasing outdoor transmission: A Qualitative study in rural Tanzania, submitted to Malaria Journal for publication**].

### Clinical data collection

A single government health facility in the study area was recruited into the study. At this facility, health services were provided for free by the project if the participants showed their project identification card with a household unique identification number on it. Community members that were not enrolled into the study were issued with a different kind of identification card to also allow them free consultation and treatment at the recruited health facility. This was done to discourage community members attending the health facility under the guise of being a study participant and, therefore, contaminating the study by recording malaria status of community members not enrolled in the study as participants. It was assumed that since services were provided for free at this facility, it would attract most community members seeking health services. A clinical officer (CO) and a nurse were employed by the project at this health facility. A ledger with the household unique identifier and names of each household member was drawn up and placed at this health facility. When a study participant visited the health facility with febrile illness, the CO checked against their name and household unique ID in the health facility ledger. This way household and health facility data could be reconciled using the household unique identifier. Febrile participants were tested for malaria using rapid diagnostic test (RDT) (ICT Malaria cassette tests HRPII/pf test kit). A proportion of participants also had diagnosis by thick film microscopy to confirm the accuracy of the RDTs for diagnosis under field conditions. The result of the RDT and the date of diagnosis were marked against the Household ID on the health facility ledger. Those found positive for malaria parasites were given artemether-lumefantrine (ALu), the first-line drug for treatment of malaria in Tanzania. Only participants that were RDT or slide positive for malaria parasites were treated. This was to avoid treating non-malaria patients with ALu, which might have affected malaria incidence rate in the village. The RDT’s were labelled with the patient’s unique identifier, date and status (+ve or –ve) and stored for verification. These were later checked against the clinical trial database to ensure that no cases had been incorrectly entered into the database by the clinic staff.

### Data management

Data from the structured questionnaires on SES of households and KAP in relation to malaria and repellents administered at baseline; follow-up data on compliance and recalled febrile illness administered throughout the study period; and the after study KAP survey, were double entered into a computer using an Epi –Info™ template with a drop down lists of values that corresponded to the format of the questionnaires. Data was then exported to Microsoft Access 2008 (Microsoft Corporation), to check for lack/excesses of data, inconsistencies and outliers. All data from the above mentioned questionnaires were linked using the household unique identifier. The household unique identifier was made up of the household number, cluster number and treatment number.

### Statistical analysis

Data was collected and presented at household and cluster level as the study aimed at assessing the effectiveness of the repellents at the community level. Individual level data was not collected.

### Socio-economic status (SES)

All data cleaning and analysis was performed using STATA 11.2 software (StataCorp LP, College Station, Texas, USA). Baseline household-level socio-economic indicators were collected using a structured questionnaire. All variables representing asset ownership, household construction materials, source of fuel and light and the education level of the household head were examined individually before being combined using principal component analysis (PCA) to generate the socio-economic index of each household, [33], and are presented in here: (Additional file 1: Stata output showing Eigen scores of each variable used in calculation of socio economic status of households). The households were grouped into quintiles of the socio-economic index generated and ranked from the poorest to the least poor. This data was cross tabulated with treatment group using Pearson’s chi-square (χ^2^) to assess whether there was a significant difference in the socio-economic status of the households in the two treatment groups (not accounting for the clustered design due to the exploratory nature of this analysis.

The number of treatment tubes issued was analysed by linear regression against month, treatment and an interaction of month and treatment to determine if there was a significant difference in the number of tubes issued in each month and per treatment group.

### Clinical data

Clinical data was adjusted for covariates identified *a priori* to be confounders and analysed using the according-to-protocol approach, where person-time at risk was excluded when a participant reported or was observed to be non-compliant to the lotion (placebo or repellent) and for those with malaria for three weeks after they were diagnosed. The total number of cases in each treatment group was divided by the sum of person years at risk to give the incidence rates in person years at risk. Rate ratio and rate differences were then estimated.

For comparison, a secondary analysis using the intention-to-treat approach, where malaria incidence rates in the clusters were compared using all person-time at risk regardless of whether they complied with the study protocol but also adjusted for covariates identified *a priori* as confounders. Such an approach would be expected to underestimate the treatment effect. It was not possible to effectively blind the PI and PL as they had carried out both the semi field and field efficacy evaluations of these treatments [19] and could identify the intervention and placebo. The clinical data was, therefore, re-blinded by an independent statistician (ET), who was not aware of the intervention and placebo codes.

### Person-time at risk estimation for according-to-protocol analysis

The study was conducted for 14 months from July 2009 to August 2010. To calculate the person-time at risk, a closed cohort was assumed, so that the number of household members above six months recorded at baseline for each household was assumed to be constant throughout the study period. Monitoring surveys were conducted for each month of the study to establish compliance.

Person time at risk of each household was estimated according to one of the following three possible scenarios:

1. In a case where all individuals were susceptible to malaria infection and complied with the study protocol by applying the treatment issued on a nightly basis, each individual in the household was assumed to contribute one-person month at risk to the study.
2. In a case where the household head or an able household member was not available to take the monthly monitoring surveys, it was assumed that all members of that household did not comply with lotion (repellent or placebo) use for that month and one-person month at risk for each member of that household was excluded from the person time at risk of the study.
3. In a case where a household member contracted malaria, that individual was excluded from calculation of person time at risk for three weeks.

Person-time for all household members was calculated according to the appropriate scenario above.

### Malaria incidence rates and regression analysis of the intervention effect

Using data on the total number of confirmed malaria cases and person-time for each household, we used a two-stage approach to estimate intervention effects (recommended by Hayes *et al.* for studies with fewer than 15 clusters/group) [27]. In the first stage, cluster-specific incidence rates were calculated using random effects Poisson regression modelling with adjustment for confounding variables. Specifically, the outcome of total number of confirmed cases of malaria/household was regressed on the set of confounding variables (age categories of the household, education of the household head, and quintile of SES), with an offset for person-time at risk per household and a random intercept for cluster to account for the clustered study design. As per Hayes *et al*., treatment was not included as a factor in the model. In the second-stage, residuals, calculated from the regression model were aggregated by clusters. The covariate-adjusted treatment effect was then estimated by comparing the residuals in the intervention relative to the control group using the Wilcoxon rank sum test, because the data were not normal.

### Knowledge attitude and practice (KAP) of community members in relation to malaria and repellent

Baseline data on knowledge of malaria and malaria prevention practices and knowledge and practice in relation to repellents were analysed using descriptive statistics in STATA 11.2 to assess whether there was an imbalance between the treatment arms. Data that recorded attitude with regards to repellents, perceived effectiveness and willingness to continue use and pay were also analysed and these results are presented elsewhere [Sangoro O, Sarah M, Ann HK, Sarah M: **Feasibility of repellent use in a context of increasing outdoor transmission: A Qualitative study in rural Tanzania, submitted to Malaria Journal for publication**].

### Ethical and safety considerations

During recruitment, the household head was asked for written informed consent for themselves and all household members. If consent was obtained, all members of the household were recruited into the study. Study participants were free to withdraw from the trial at any time. All households in the village were issued with an LLIN for every sleeping space to ensure equity. All individuals from the study village were allowed free consultation, treatment and drugs (ALu) from the village dispensary at project cost. Participant confidentiality was maintained by using generated unique identifiers instead of individual names during analysis.

Participants were educated on correct repellent use and application. Children under 6 months were excluded from the trial. An illustrated label giving instructions in the native language (Swahili) on safe repellent use was provided on each tube. DEET repellent used in this study has undergone extensive toxicological tests and has been endorsed as safe for human use [32]. The concentration of DEET (15%), used in this trial was approved by the Tanzanian Pesticides Research Institute, the Tanzanian Bureau of Standards and is available in Tanzanian shops. Guardians to children < six months were reminded to put their children under an LLIN early to prevent them contracting malaria. A clinical officer (CO) was employed at the village dispensary by the project to perform RDTs and to investigate and treat any adverse effects arising from repellent use.

Ethical approval for the study was obtained from Ifakara Health Institute (IHI) (IHRDC IRB A46), Tanzanian National Institute of Medical Research (NIMR/HQ/R8a/VOL IX/780) and the London School of Hygiene and Tropical Medicine Ethical Review Board (LSHTM ERB 5174). IHI provided study monitoring.

## Results

### Trial profile and baseline data

**Figure 2:**
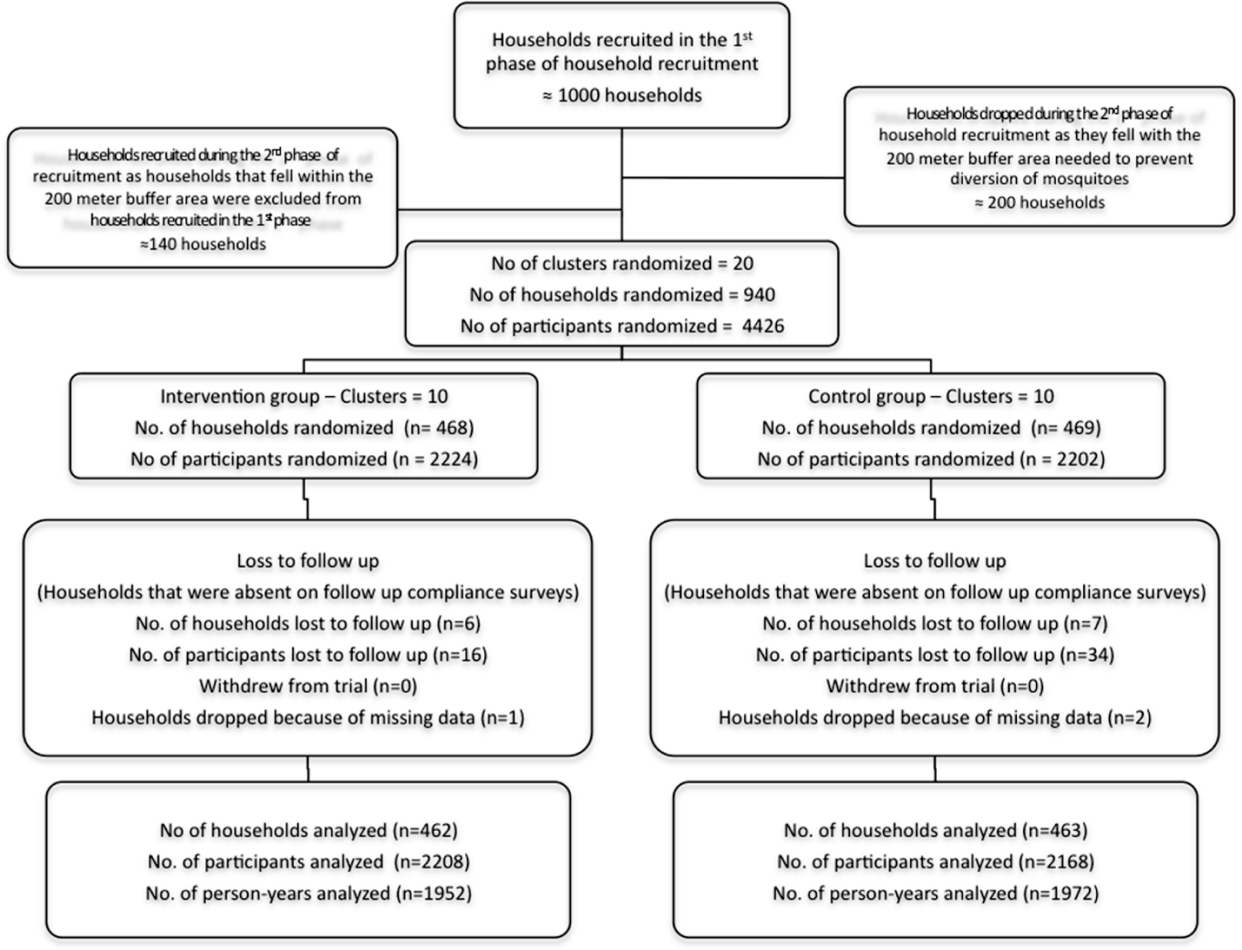
**Trial Profile.**

The trial profile is summarized in Figure 2. In the intervention group 2,224 individuals were enrolled and 2,202 in the placebo group. Loss-to-follow up was higher in the placebo group: n = 34 versus n = 16, and no individuals withdrew from the trial. Similar numbers of person-years were analysed: 1952.81 in the intervention group and 1972.38 in the control group of the trial. Baseline household level socio-economic data on education and gender of household head, age-groups of all study participants, household construction material, source of cooking fuel and lighting and asset ownership were examined individually and are presented in Table 1. The gender of the household heads was comparable between the two treatment groups, with 55.33% (n = 514) females and 44.67 (n = 415) males. Most of the household heads had received some form of formal education, 82.81% (n = 702) while only 17.18% (n = 161) had no formal education. Of all participants recruited in the study, 17.55% (n = 771) were children under five years of age, 34.37% (n = 1,510) were between five to 18 years of age and 48.08% (n = 2,112) were above 18 years of age and age-category distribution was similar in the two treatment groups. The predominant source of energy used by the households was wood fire, 89.96% (n = 883), while the predominant source of lighting used was the traditional lamp, 93.76% (n = 871). Assessment of household construction materials demonstrated that most households in the study area had floors made from mud, 82.78% (n = 769), while tin and thatch were used equally as roofing materials, 49.35% (n = 457). Also, most households in the study area had walls made from bricks, 79.87% (n = 742). Socio-economic indices generated from PCA suggested an imbalance between the two treatment groups, with the control group demonstrating a higher SES than the intervention group, (Pearson’s χ^2^ = 17.5519, p = 0.002), (Table 2).

**Table 1:** **Baseline household characteristics by treatment group**

	Intervention arm n (%)	Control arm n (%)	Totals n (%)
No. of households	469 (50.05)	468 (49.95)	937 (100)
No. of participants	2224 (50.05)	2202 (49.95)	4426 (100)
*Gender of household head*			
Male	215 (46.24)	200 (43.10)	415 (44.67)
Female	250 (53.76)	264 (56.90)	514 (55.33)
*Education of household head*			
No education	83 (17.74)	78 (16.63)	161 (17.18)
Educated	385 (82.26)	391 (83.37)	702 (82.82)
*Age group distribution of all participant/household*			
Under 5’s	412 (18.50)	359 (16.57)	771 (17.55)
5-18 years	721 (32.38)	789 (36.43)	1510 (34.37)
Above 18 years	1094 (49.12)	1018 (47.00)	2112 (48.08)
*Source of energy*			
Wood fire	431 (92.89)	402 (86.83)	883 (89.86)
Other sources	33 (7.11)	61 (13.17)	94 (10.14)
*Source of lighting*			
Traditional lamp	445 (95.70)	426 (91.81)	871(93.76)
Other source	20 (4.30)	38 (8.19)	58 (6.24)
*Flooring material*			
Mud	404 (86.88)	365 (78.66)	769 (82.78)
Cement	61 (13.12)	99 (21.34)	160 (17.22)
*Roofing materials*			
Thatch	256 (55.41)	201 (43.32)	457 (49.35)
Tin	203 (43.94)	254 (54.74)	457 (49.35)
Other	3 (0.65)	9 (1.94)	12 (1.30)
*Wall materials*			
Mud	121 (26.08)	66 (14.19)	187 (20.13)
Bricks	343 (73.92)	399 (85.81)	742 (79.87)
*Assets ownership*			
Motorbike			
Yes	72 (15.48)	52 (11.18)	124 (13.33)
No	393 (84.52)	413 (88.82)	806 (86.67)
Bicycle			
Yes	246 (52.90)	198 (42.58)	513 (55.16)
No	219 (47.10)	267 (57.42)	417 (44.84)
Stove			
Yes	344 (73.98)	314 (67.53)	658 (70.75)
No	121 (26.02)	151 (32.47)	272 (29.25)
Mobile phone			
Yes	197 (42.37)	211 (45.38)	408 (43.87)
No	268 (57.63)	254 (54.62)	522 (56.13)
Radio			
Yes	140 (30.11)	156 (33.55)	296 (31.83)
No	325 (69.89)	309 (66.45)	634 (68.17)

**Table 2:** **Ranking of households using Socio-economic scores generated for PCA analysis by treatment group**

	Intervention arm n (%)	Control arm n (%)	Total n (%)	Pearson’s Chi2	P value
***SES generated from PCA***					
Poorest	39 (8.33)	28 (5.97)	67 (7.15)		
Poor	164 (35.04)	121 (25.80)	285 (30.42)	17.5519	0.002
Median	165 (35.26)	174 (37.10)	339 (36.18)		
Less poor	77 (16.45)	107 (22.81)	184 (19.64)		
Least poor	23 (4.91)	39 (8.32)	62 (6.62)		

The use of repellents as a mosquito control tool was low in the study area, with only 1% (n = 6) of those interviewed reporting to have ever used repellents. Results on KAP of repellents are presented in details elsewhere [Sangoro O, Sarah M, Ann HK, Sarah M: **Feasibility of repellent use in a context of increasing outdoor transmission: A Qualitative study in rural Tanzania, submitted to Malaria Journal for publication**].

The average number of tubes issued per household was 6.73 (95% C.I. 6.51 – 6.95) and 6.92 (95% C.I. 6.68 – 7.16) in the intervention and control group respectively and there was no significant difference per treatment group, 1.68 (95% C.I. 0.32 – 84.25, P = 0.803) from linear regression analysis. Likewise there was no significant difference on the number of treatment tubes issued per month throughout the study period.

### Clinical outcomes

#### According-to-protocol analysis

When data was analysed as per protocol there was a non-significant difference in cluster and household malaria incidence rates among repellent users and non-users (Table 3). In the cluster-level analysis (data averaged over cluster specific rates), the malaria incidence rates differed by 11.48%; with 68.29 (95% C.I. 37.05-99.53) cases/ 1,000 person-years in the control group and 60.45 (95% C.I 48.30-72.60) cases/1,000 person-years (95% C.I. 44.55 – 81.73) in intervention group, (Wilcoxon rank sum z = 0.529, p =0.5967). For household-level malaria incidence rates (data averaged separately over household specific rates), the incidence rates differed by 28.88%: with 84.54 (95% C.I. 61.04-108.05), cases/1,000 person-years in the control group and 60.12 (95% C.I. 45.08-75.15) cases/1,000 person-years in the intervention group, (Wilcoxon rank sum z = -1.267, p = 0.2051). These result should however be interpreted with caution as there is still an ongoing debate on whether it is correct to estimate incidence rate ratios using regression models on less than 10 clusters [28]. Cluster aggregated rates were reported because it measured the overall effect of the intervention at the population level [34] and this was the major objective of the study. Age was a significant risk factor with risk decreasing with increase in age. SES did not influence the risk of malaria in the model.

**Table 3:** **Estimated incidence rates by treatment arm and estimated intervention effects**

	Intervention arm	Control arm	% Reduction in rates	Wilcoxon rank-sum on residuals (p-value)
Malaria cases	115	137		
**ATP analysis**				
Individuals randomized	2208	2168		
Households randomized	463	462		
Total person-years	1952.81	1972.38		
Average Household rates/1000 person-years	60.12 (95% C.I 45.08-75.15)	84.54 `(95% C.I 61.04 108.05)	24.42%	-1.267 (0.2051)
S.D.	164.42	257.07		
Average cluster rates/1000 person-years	60.45 (95% C.I 48.30 72.60)	68.29 (95% C.I 37.05-99.53)	8%	0.529 (0.596)
S.D.	16.98	43.66		
**ITT analysis**				
Individuals randomized	2224	2202		
Households randomized	468	469		
Total person-years	2580.44	2554.92		
Household rates/1000 person-years	47.26 (95% C.I. 35.49-59.04)	68.21 (95% C.I. 49.59-86.84)	20.95%	-1.268 (0.2047)
S.D.	129.60	205.23		
Cluster rates/1000 person months	45.43 (95% C.I 36.02–59.79)	53.21 (95% C.I. 30.98–104.16)	7.78%	0.227 (0.8206)
S.D.	11.32	34.90		

### Intention-to-treat analysis

Cluster-level analysis of malaria rates in the two treatment arms demonstrated a non-significant, 14.62% difference in malaria rates with 53.21 cases/1,000 person-years (95% C.I. 30.98 – 104.16) in the control group and 45.43 cases/1,000 person-years (95% C.I 36.02 – 59.79) in the intervention group, (Wilcoxon rank sum z = 0.227, p = 0.8206), (Table 3). Household-level analysis of malaria incidence rates demonstrated a 30.71% difference in malaria incidence rates, with 68.21 cases/1,000 person-years (95% C.I. 49.59 to 86.84) in the control group and 47.26 cases/1,000 person-years (95% C.I. 35.49 – 59.04), in the intervention group, (Wilcoxon rank sum z = - 1.268, p = 0.2047). Age was a significant risk factor: malaria risk decreased with increase in age although SES did not influence the risk of malaria in the model.

## Discussion

This randomized controlled trial demonstrated that 15% DEET topical repellents have no effect on malaria incidence transmitted in the early evening. Although there was a consistent decrease in malaria risk among repellent users in both the cluster and household malaria rates, as seen from the results above, this reduction was not significant. This finding is consistent with a study carried out in southern Lao PDR using an identical 15% DEET repellent [35]. It should be noted that, findings from other studies using a higher concentration of 20% DEET with Permethrin in soap that gave over 12 hours of complete protection from mosquito bites [11] and Para-menthane 3–8 diol repellents with close to 100% efficacy for over six hours [30, 36] did demonstrate a significant protective effect in Pakistan [11], Bolivia [10] and Ghana [23] and this could be one of the potential explanations for the observation of a treatment effect in these studies. It can be argued that in the Lao-PDR study, 15% DEET provided ~ 100% protection against mosquito bites. However, the number of major malaria vectors, *Anopheles minimus* and *Anopheles maculatus,* caught in entomological collections in the Lao-PDR study was very low and that the effect observed, was probably that of 15% DEET against *Stegomyia* and *Culex* mosquitoes which made up the bulk of the collections. Therefore, as Anophelines are known to show less response to repellents compared to *Stegomyia* and *Culex* mosquitoes [37, 38], the repellent effect observed in the Lao-PDR study was greater than at higher densities with a greater proportion of Anophelines as tested in Tanzania [19].

### Power

There are several factors that are likely to have masked any treatment effect in this study, the most likely being the lack of power to discriminate a statistically significant difference between study arms. The lack of power in the study was likely caused by four factors:

First, rapid scale-up of LLINs to achieve universal coverage has been actively taking place in Tanzania [16]. This had led to a substantial decline in malaria in the country and by extension the study area [39]. As a result, the incidence of malaria in the village was likely lower than the incidence assumed for calculation of sample size for this study. This likely led to an underestimation of the sample size required to observe a difference between the two treatment groups. Secondly, during the study period, Tanzania experienced a drought that likely further reduced malaria transmission, and as a result, there were too few malaria episodes in the study area to accurately discriminate any reduction in malaria attributable to the repellent [40], highlighting the need for such studies to be carried out for more than one transmission season to avoid such problems. Third, most of the participants recruited in to the study come from a farming community. Therefore, during the planting and harvesting seasons, these participants relocated to their farmhouses [41]. As a result it was difficult to establish compliance during these periods and those participants were excluded from the study. This lowered the study sample size further and with it the power to detect a treatment effect. Lastly was the likely overestimation of the assumed malaria incidence in the study area that was used for sample size calculations. Malaria incidence in this study was estimated from reported fever rates in children less than 5 years of age in the study area [26]. Therefore, even though scale up of LLINs and the drought experienced during the study might have lowered the malaria incidence in the study area, it is also likely malaria rates used for estimation of sample size might have been overestimated and hence undermined the study power to observe a difference between the treatment groups.

### Compliance

Compliance in this study was measured by self-reporting of use every evening by the household head or a household member that was able to engage with the field workers during the monitoring surveys. However self-reporting is an unreliable measure of compliance, as it have been shown to overestimate compliance [42]. As a result, the ATP analysis used to measure malaria incidence is likely to underestimate the actual malaria incidence in the intervention and control arms, as a larger value of person-time will be used than that of individuals that actually complied to the study reducing discriminatory power. However, if the randomisation between the two treatment groups was done correctly then the overestimation of compliance and its resultant effect of the study outcome, is likely to be similar in both treatment groups, ruling out the likelihood of overestimation of the treatment effect. This underlines the importance of correctly estimating the compliance in studies of personal protection in order to avoid confounding the outcomes of such studies.

### Active versus passive case detection

Due to logistical reasons, this study recruited a single government health facility for collection of clinical data by passive case detection. As a result, the study is likely to have lost malaria cases to the other health facility present in the area. Anecdotally, some participants complained that they went to the other health facility because the study facility always told them that they did not have malaria even though they knew they had malaria, so they did not trust the diagnosis. Also some individuals might have opted to use traditional medicine, treat diseases at home or buy drugs directly from the numerous drug stores in the study area if they felt sick. All these are potential malaria cases that the study might have lost, lowering both the sample size and estimates of malaria incidence in the area. It would have been advantageous to collect data from both health facilities or carry out active case detection. Since malaria was still most common in children under five years in the study site as seen elsewhere [43, 44], targeted active case detection in under fives may have gathered more reliable and realistic data on the true impact of repellents in this scenario. Performing supplementary testing of blood spots from all participants attending the health facility with polymerase chain reaction (PCR) diagnosis of subclinical malaria parasitaemia may have also yielded more accurate estimation of transmission prevention by repellents [45].

### Sources of bias

Bias was introduced into the study by an imbalance in socio-economic status between the two study groups. The control group demonstrated a higher socio economic status than the control arm. This study however, did not demonstrate a statistically significant association between SES and malaria incidence. However, it is well known that improved housing, whose representative covariates had been adjusted for during analysis, is protective against malaria [46]. A plausible explanation for this is that the participants in this study came from a single village or from villages located closely together. As result they were exposed to the same levels of malaria transmission regardless of their socio-economic status. As socio-economic status is positively associated with seeking treatment at a medical facility [47], it is likely that participants with higher SES sought treatment at the health facility in the study area at a higher rate compared to participants in the lower SES. Therefore as malaria data was only collected from a single health facility, it is likely that more cases of malaria were observed in participants with higher SES relative to participants from lower SES. Another reason is that no association was seen may be because studies using material ownership as a proxy for measuring SES, to evaluate the relationship between SES and malaria incidence have yielded inconsistent results, at the household level [48].

The study participants were blinded up to some point after allocation of treatments, because of the identical packaging labelled with a three-digit code. However, after a while, field workers reported that study participants in the placebo group complained that they wanted to swap treatment. Participants could differentiate the intervention from the placebo, as mosquitoes would still bite them after applying the ‘treatment’ while those in the treatment group bragged to their neighbours that they got the good lotion that was effective. This is a source of bias and could have caused treatment contamination between clusters. This problem would have been better overcome with clusters that were geographically isolated, for instance randomization on a village scale, so that individuals were less likely to be able to compare their treatment allocation. Some participants may have sold or given their repellent to relatives in other clusters.

Another potential confounder may have been diversion of mosquitoes from the intervention group to the placebo group. However, this was controlled by allowing for a buffer area of 200 metres between clusters. Diversion in repellent studies has usually been recorded over short distances, one metre [30]. However, distances of 15–20 metres are recommended as the limit for short range attraction of host seeking mosquitoes [49, 50] and, therefore, distances of 200 metres between clusters were thought to be adequate to prevent diversion. Treatments were also issued at the household level to prevent intra and inter-household diversion within the cluster. It has been later observed in the study area that mosquito diversion between households does occur [29] and could have confounded data if compliance with the intervention was low by diverting mosquitoes from complying to non-complying households or individuals.

The community was highly knowledgeable about malaria transmission, prevention and control. This is likely a result of the malaria intervention programmes that have taken place in the study village for over two decades [14, 17]. The community awareness about topical repellents as a mosquito control tool was poor at the study inception. However, after the study, the community was highly aware of repellents and community members were willing to take up this intervention against malaria if available. This finding demonstrates the feasibility of topical repellents as a potential tool to supplement LLINs to prevent early evening transmission. In a separate study [Sangoro O, Sarah M, Ann HK, Sarah M: **Feasibility of repellent use in a context of increasing outdoor transmission: A Qualitative study in rural Tanzania, submitted to Malaria Journal for publication**], the community members reported bite avoidance as the major reason for using repellents in the early evenings.

*A posteriori* analysis of data for children under six months was carried out to check whether this age group experienced high malaria transmission because of mosquitoes diverted to them as it was recommended that they not use the repellent [29, 30]. This might also have affected the incidence of malaria in the treatment groups if there was uneven distribution of this age category between these groups. However, it was observed that there were only three children and a single case of malaria in this age category, and it can be confidently concluded that this age group did not have any influence on the outcomes observed.

Net usage was also analysed to determine whether there was a difference between the two treatment groups, which would have confounded the outcome. It was observed that reported net usage the previous night was 100% in both treatment groups. These results are presented in detail elsewhere [Sangoro O, Sarah M, Ann HK, Sarah M: **Feasibility of repellent use in a context of increasing outdoor transmission: A Qualitative study in rural Tanzania, submitted to Malaria Journal for publication**].

### Recommendations

It was observed that estimation of a sample size with sufficient power was a major shortcoming of this study. Therefore, it is advisable to establish baseline disease incidence rates if a similar study is to be implemented in the future to avoid under powering the study. This can be established from health facility records. However these records may not necessarily be accurate and the more appropriate measure may be to conduct a small cross-sectional or longitudinal survey of the community disease prevalence or incidence and then power accordingly. Another important factor when testing personal protection tools is accurate establishment of compliance. Better methods of establishing compliance are needed. This can be done through frequent follow-up and spot checks or use of indirect methods, such as mosquito saliva antigens, that are a proxy of individual exposure to mosquito bites [51]. Also, development of new tools that require reduced compliance such as long lasting spatial repellents [52] would likely offer greater protection because people often forget to comply daily with a topical repellent unless they feel mosquito bites [53]. Finally, in a time when malaria is becoming more scant due to successful control, active case detection using RDT for clinical diagnosis followed up by PCR for malaria parasites is most likely the most appropriate means of measuring the impact of additional malaria control tools used in combination with LLINs.

## Conclusion

Findings of this trial could not demonstrate if 15% DEET topical repellents had any impact on incidence of malaria transmission in the early evening because the study lacked sufficient statistical power and had several important sources of bias. A better-designed study with sufficient power and fewer sources of bias and ideally a higher concentration of repellent is required to fully understand if topical mosquito repellents are a feasible malaria control tool in the early evenings in Eastern Africa, particularly as repellents have reduced malaria elsewhere in sub-Saharan Africa [23, 24]. The acceptability of this intervention is an encouraging finding toward exploring supplementary malaria control tools.

## Electronic supplementary material

Additional file 1: **Stata output showing Eigen scores of each variable used in calculation of socio economic status of households.** (PNG 120 KB)
