# Supplementary figures and images for: A cluster-randomized controlled trial to assess the effectiveness of using 15% DEET topical repellent with long-lasting insecticidal nets (LLINs) compared to a placebo lotion on malaria transmission
Source: Malar J. 2014 Aug 16;13:324. doi: 10.1186/1475-2875-13-324 (PMC4247706; doi:10.1186/1475-2875-13-324)

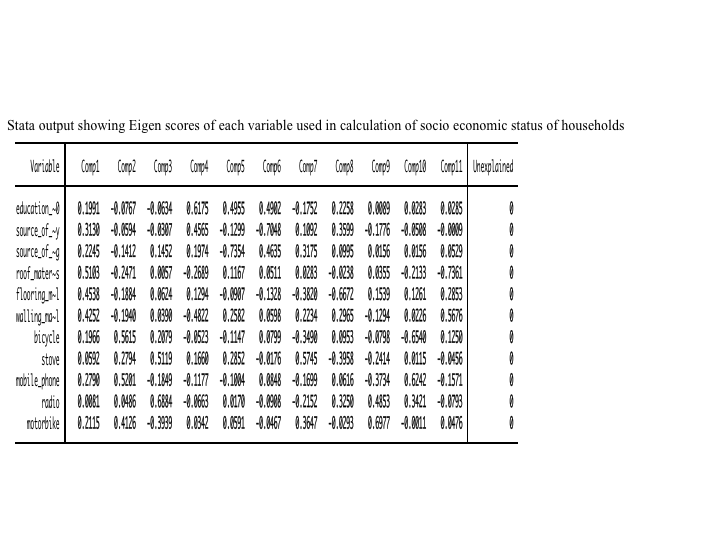

Supplement: Supplementary file 1 — Additional file 1: Stata output showing Eigen scores of each variable used in calculation of socio economic status of households. (PNG 120 KB) [file 12936_2014_3601_MOESM1_ESM.png]
